# Experiences of caregiving with Alzheimer’s disease in the LGBT community

**DOI:** 10.1186/s12877-023-03914-1

**Published:** 2023-05-15

**Authors:** Carey Candrian, Emily S. Burke, Danielle Kline, Alexia M. Torke

**Affiliations:** 1 Division of General Internal Medicine, University of Colorado School of Medicine, 12631 East 17th Avenue, Aurora, CO 80045 USA; 2 Regenstrief Institute, Indiana University Center for Aging Research, Indianapolis, IN 46202 USA; 3 Division of General Internal Medicine and Geriatrics, Indiana University Center for Aging Research, Regenstrief Institute, Indiana University, Indianapolis, IN 46202 USA

**Keywords:** LGBT caregivers, LGBT older adults, Health disparities, Surrogate decision making, Phenomenology

## Abstract

**Background:**

The goal of this paper is to develop a more thorough understanding of the experiences of LGBT older adults living with dementia and their caregivers.

**Methods:**

A phenomenological approach using in-depth interviews with current or former caregivers of LGBT persons living with Alzheimer’s disease (AD) was conducted.

**Results:**

Participants ranged in age from 44–77 years old; 74% were lesbian, 16% gay, 5% straight, and 5% unknown. Five themes were identified from the analysis: Caregiver tension and isolation; financial stress & security; lack of social support & connection; engineering grief support, and entrapment of past and present stigma and discrimination.

**Conclusions:**

Discrimination related to LGBT status was an important theme over the participants’ lives and occurred for several during dementia care. While other themes were similar to prior AD studies, LGBT status affected these other aspects of the caregiving experience. Findings can inform future programs that better meet needs of LGBT people and those who care for them.

## Background

In 2022, an estimated 6.5 million Americans aged 65 and older are living with Alzheimer’s disease and related dementias (ADRD); by 2050, the number of people aged 65 and older with ADRD is projected to reach 12.7 million [1]. Women comprise 2/3 of ADRD cases and the majority of caregivers [2]. By 2020, this number could increase to 16 million. One particular population who face a particularly challenging set of circumstances when they receive a dementia diagnosis are lesbian, gay, bisexual, transgender and queer (LGBT) older adults. An estimated 200,000 LGBT people currently live with dementia [3]. As the number of people living with ADRD continues to grow, so does the number of Americans who will take on a caregiving role.

Older LGBT adults have faced a long history of structural discrimination leading to health disparities. LGBT people have higher rates of depression and higher rates of alcohol, tobacco use [4, 5], certain cancers [6, 7], and cardiovascular disease [8–10]. Additionally, risk factors for poor health outcomes in LGBT older adults both increase their likelihood of receiving an Alzheimer’s diagnosis and increase the challenges faced by caregivers for someone with ADRD. Risk factors for heart disease – including diabetes, tobacco use, high blood pressure and high cholesterol – are also risk factors for ADRD [6]. Fifty six percent of LGB and 70% of transgender older adults reported being denied or provided inferior health care because of their gender identity [9]. As a consequence of discrimination, many LGBT older adults have hidden their identity and relationships for decades; 48% of older LGBT adults said their physician does not know they are LGBT [10]. These numbers underscore how past experiences of discrimination and fear influence interactions with or avoidance of the healthcare system and other services [11]. These risk factors, including the stress of hiding one’s identity and the effects of structural discrimination can take up to 12 years off of LGBT lives [12].

LGBT people who receive a dementia diagnosis are twice as likely to live alone, four times less likely to have children, and more likely to be estranged from family and peers. They are also more likely to face poverty and homelessness, and to have poor mental and physical health outcomes when compared to heterosexual populations [4, 5]. LGBT adults also experience stigma and discrimination at a much higher rate than their heterosexual peers [7, 9–14]. Prior discrimination has been shown to shorten lifespan and is associated with worse mental health. Discrimination when seeking care also has the potential to reduce the quality of care for the person with dementia and increase caregiver strain [13].

On average, caregivers of people with ADRD provide care for longer periods of time, than do caregivers of older adults with other conditions [1]. LGBT people become caregivers at a higher rate than the general population [10]. This may lead to long term inequities between LGBT communities and ADRD in terms of support and other services.

Much is known about the costs of caregiving in terms of psychosocial and physical health effects. Dementia caregivers report higher levels of stress, more depression and anxiety symptoms, lower levels of well-being, self-efficacy, and anxiety. On top of that, they experience worse physical outcomes: higher levels of stress hormones, compromised immune response, antibodies, greater medication use, and greater cognitive decline. Spouses, women, and those in lower socioeconomic groups appear more vulnerable to these health outcomes. Although more than 200,000 LGBT people are living with dementia, the experiences and needs of LGBT people living with dementia and their caregivers have not been adequately understood, interpreted, or addressed. A previous study from 2016 illuminated the psychosocial factors in older lesbian, gay and bisexual people including societal stigma and the duality of managing dementia and disclosing your identity [15, 16]. A more recent study in 2021 identified characteristics of LGBT caregivers of people with dementia given what little is known about their experiences [17]. Although a majority of their sample were gay men, the study was the first to provide a focused description of the characteristics and psychosocial factors associated with depressive symptoms and quality of life in the caregivers. Because a lot of the protective factors for caregiving (e.g., social networks, knowledge/information, economic, community resources, and other vulnerabilities) are the very things many LGBT older adults lack, more research in this area is vitally important. The goal of this paper is to develop a more thorough understanding of the experiences and needs of LGBT older adults living with Alzheimer’s disease (AD) and their caregivers.

## Methods

### Theoretical framework

This study seeks to provide an understanding of the intersection between AD caregiving and the LGBT experience. This investigation was guided by phenomenology [18, 19] that focuses on the assessment of individuals’ narratives in order to understand what those individuals experience in their daily lives – in this case, caregiving for those living with AD as a sexual minority. The first step in this process is to identify an interesting phenomenon (i.e. the experience of LGBT caregivers, including constructs of voice and identity). The research team then investigated the experience of caregivers for LGBT older adults as it is actually lived through review of the individuals’ own voice, experience, and identity as a caregiver. Our team captured themes as they were presented by participants in a cycle of writing and reflecting, creating a continuous and iterative cycle to develop a nuanced and rich analysis. Throughout, we maintained a strong orientation to LGBT caregiver experience while attending to the interactions between the data (the parts) and how they contribute to the larger phenomena of LGBT caregiving (the whole). Although underutilized in health care, phenomenology – particularly hermeneutic phenomenology – focuses on the interpretation of these experiences and is a novel methodology for understanding a phenomena from the perspective of those who have experienced it [19, 20].

### Design

Qualitative semi-structured interviews were conducted with caregivers of LGBT older adults from various cities in the United States.

#### Recruitment strategy

Caregivers of LGBT older adults were recruited across the United States using a combination of random and snowball sampling techniques. Two members of the research team contacted over 75 community organizations, LGBT support groups, memory clinics, Alzheimer’s Association chapters, as well as community leaders, advocates, and practicing clinicians and asked them to distribute information about the study. Participants then contacted the research team directly if they wished to participate and were provided with consent documents. Participants gave verbal consent that was approved by the Indiana University Institutional Review Board (Protocol # 1,908,440,258).

Participants were interviewed by one of two researchers. We checked comprehension of responses with each participant throughout the study and at the end of each interview to ensure accuracy of data collected. At the end of each interview, participants were asked to invite other individuals they knew who might participate and provide the names of other organizations who may have eligible participants.

#### Data collection

We conducted 19 in-depth interviews with caregivers aged 44–77 (median: 68.50, mean: 66.94) over a 9-month period from December 2019 to September 2020. Caregivers were 79% female, 89% white, and 32% Latino/Hispanic. Sexual orientation was 74% lesbian, 16% gay, 5% straight, and 5% unknown. We did not intentionally exclude transgender people, but we were unable to recruit these participants for this study, which is a limitation that we address later. Among the varied research approaches to assess saturation, a 2022 in line qualitative systematic review converged on a relatively consistent sample size for saturation: using empirical data saturation was reached within a narrow range of interviews (9–17). In line with this, we reached saturation after 19 interviews [21]. Over half of the participants (57%, *N* = 11) were married to a same sex partner, while 32% (*N* = 6) were with a same sex unmarried partner. The majority of caregivers were spouses of the person with AD (53%, *N* = 10) and 26% (*N* = 5) were identified as spouse equivalent/unmarried partners. Approximately 68% of the caregivers had been in a relationship with the person living with AD for 15 years or more. Participants were from 8 states in demographically diverse regions of the United States (e.g., California, Colorado, Georgia, Maryland, Connecticut, New Mexico, Minnesota, and Missouri).

Five interviews were conducted face-to-face before the COVID-19 pandemic, with the remaining interviews conducted by phone or virtual meeting during the pandemic (14 interviews). Interviews were conducted and audio-recorded by the co-principal investigator (11 interviews) and a research assistant (8 interviews) and lasted 20–60 min. The majority lasted around 60 min. Interview questions were designed by the research team and then reviewed by palliative care groups. Questions covered topics that explored how caregivers understand their role and how they manage the tasks associated with caring for the person living with AD. Study data were managed using REDCap (Research Electronic Data Capture) hosted at the University of Colorado [22].

#### Data analysis

Interviews were professionally transcribed and deidentified, yielding 248 pages of raw data to be analyzed. Transcripts were coded by using the constant comparative method [23] with an inductive data coding process used to categorize and compare qualitative data for analysis. To avoid bias in the analysis, multiple members of the research team coded the data separately to gather alternative explanations from each researchers’ positionality: social scientist, physician, and two research professionals.

Four members of the research team read every interview and independently coded the data. We developed an initial codebook based on the first few interviews but continued to add codes throughout the analysis process as new themes were identified (via the codebook). The research team met twice per month for 6 months to review codes, discuss recurring themes, and write memos [24]. Codes and themes were refined further during team discussions, which aided the development of the codebook. We sought to adhere to the principles of phenomenology, to deeply describe and understand the experience from the perspective of the research participants. Relationships and patterns across themes were explored and then integrated with the data to develop an understanding of the intersection between caregiving, AD, and LGBT status.

## Results

Caregivers for LGBT people living with AD described stresses and strengths in several life domains. Some caregivers provided information about how LGBT status directly affected the caregiving experience in good and bad ways. Through our inductive analysis five major themes were identified: Caregiver tension and isolation; financial stress & security; lack of social support & connection; engineering grief support, and entrapment of past and present stigma and discrimination.

### Theme 1: caregiver tension & isolation

Caregivers described the strain they experienced and also their efforts to cope. One caregiver who lived with their spouse with dementia shared:

*I’m still working two days a week by phone…with clients and I get out to walk every day so, you know, I mean I’m OK. The whole situation is horrible, and I feel really bad. I mean this is not quality of life.*

Others expressed that the length of the disease had contributed to the depth and breadth of isolation they experienced:

*I thought because he was so bad the first few months that it was going to be like maybe two years or something. Now it’s eight years and not sort of the retirement I envisioned. Fortunately, I tend to be an optimist and an up person by nature. I wouldn’t consider myself depressed but sometimes it does get to you, you know?*

Similarly, another caregiver said:

*I’ll be honest. This requires a lot of patience and everybody that I talk to says yeah, you’re going to get frustrated and you’re going to get angry, and you just have to walk away…I’ve been through a lot of shit in my life, and this is the first time that I’ve ever been on antidepressants.*

The vulnerability of social isolation impacted caregiver well-being in a variety of ways. One described additional mental health challenges as well as physical ones.

*I have to take that time for myself. The one thing that I really did not do very well this past year was exercise and I’ve put on like 30 pounds and that apparently is normal, and I need to get out of that habit. I used to ride my bike at least 100 to 150 miles a week and this summer I barely rode my bike at all. Not because I couldn’t. I mean I could have left her alone for two hours while I did a bike ride, but I felt badly leaving her alone.*

Another described the exhaustion of social isolation like this:

*It’s sort of like you have a vessel that has a ton of holes in it and you’re just trying to like put as much water in as possible and it’s constantly leaking out and you know one day you’re going to lose the battle.*

Caregivers described coping strategies such as psychotherapy and the value of supportive friendships. One participant, whose partner had recently moved into a memory community, shared:

*I have my own therapist. I have one really close friend who I talk to every night which is great grounding during this period of isolation (due to COVID). It was really hard getting used to living alone again…it’s been 38 years.*

### Theme 2: financial stress and insecurity

Participants described challenges related to financial insecurity. One caregiver said:

*I mean if you can’t afford these places then, you know, what’s left is that you are doing all the caregiving at home or you’re hiring somebody to come in which could end up being just as expensive.*

Another described the challenges of maintaining their own job, while navigating the person with ADRD’s healthcare needs:

*Financially I mean it was starting to take a toll and mentally it was taking a toll because I mean I had to be there every minute talking to doctors and all that because she couldn’t always answer the question. And I didn’t always get the information back that I needed, the correct information, so I was there at the hospital all the time. I mean I’m surprised I didn’t lose my job but they were very understanding. I used up all my annual leave.*

For one couple, financial burdens impacted a decision to get married in order to remain financially stable:

*We are not married because the state covers the cost of his HIV meds which is over $50,000 a year. If we were legally married, my income would be taken into account and it would cost us too much.*

Our data also revealed the difference financial security can make:

*It’s…you know, it’s hard, it’s depressing, it’s overwhelming…it’s everything that the Alzheimer’s Association said it would be but at the same time if you have the financials behind you and you can afford help…I can afford putting in an elevator. I can afford to put in bathrooms. I can afford to have a maid come in.*

Financial standing influenced the type of support and help caregivers could afford, and influenced what options existed when considering moving into an assisted living community or seeking in-home help. Financial barriers to caregiver support negatively impacted other aspects of caregiver well-being.

### Theme 3: lack of social support & connection

LGBT older adults often do not have adult children to rely on for caregiving. Often, it is their partner or a friend, who may be age contemporaries for the person for whom they are caring. They are often dependent on informal caregivers to provide in-home support and assist with activities of daily living. One participant shared what it was like without family or other people to rely on:

*I mean when you live with somebody 24/7 there is no escape…the other thing is that we have no family here so it’s not like I can ask a sister or somebody to come over and just be here and give me a little relief. She (partner) does not want to be alone with other people. She’s at that stage where I don’t want a babysitter. ‘Don’t get me a babysitter,’ so it makes it really hard for me to ever find time to re-charge.*

Another expressed the normality of not having any support:

*Other than the person I hired to come, there really hasn’t been anybody else. We do not have a large circle of friends here because she is not very social.*

Several caregivers expressed some deficit in their supportive networks including one who shared how stigma from her family impacted the support she received while caring for her partner:

*My mom is a born-again Christian, and she is not…very accepting of me and never… of my partner so she doesn’t help at all.*

Another caregiver described how their caregiving role had isolated them from their peers:

*I have given up a lot of friends because I simply…they don’t understand, and I feel like I’m talking to a brick wall…that’s my main support. And my partner’s family, they don’t want to hear anything about it, they don’t want to know about it.*

Additionally, the role of support beyond family of origin is underscored, as one caregiver describes their robust support network of friends:

*I don’t know why I’ve been so lucky, but we have a great support network of friends. I’ve got probably I’d say five that I can count that I could call right now and say I need your help and they’d be here in five minutes. And then to add to that I’ve probably got another hundred people that might not be here in five minutes, but they would stop and do what they could to help as soon as they could. I have a wonderful whole group of people that I do Zumba three days a week with…that’s part of my extended family and there’s probably 40 or 50 people there.*

For some support groups had a positive impact on caregivers’ support, especially when they were available and intentionally inclusive of diverse identities. One caregiver explained:

*What’s been helpful is that there is a support group and when I joined that support group there was one other lesbian in the group whose partner is on a different floor…so that saved me. I mean that was incredible…the support group and nobody has any problem with my being a lesbian.*

Although support groups exist that are designed to be inclusive, finding these groups – in the community or virtually – is challenging as one participant explained:

*I would love to have a lesbian support group…but I don’t know of any way to start a lesbian support group. I mean I don’t know any other lesbians who have this problem and that’s…that’s one of the things that makes it so isolating.*

### Theme 4: engineering grief support

The lack of support and the often-long duration of ADRD increases vulnerability to grief and bereavement for caregivers. Isolation and diminished support have significant impacts on caregivers:

*It’s weird because it’s another one of those kind of silent conditions like I mean you look at her and you have no idea that she’s got Alzheimer’s and, you know, people talk to me and they have no idea how stressful this is and so…it’s really frustrating.*

Often caregivers expressed that it was difficult to share their feelings around losing a partner or best friend to a disease that is often “hidden” and misunderstood by friends, exacerbating the isolation they experience during a time when support could help buffer their feelings of grief and fear. One participant explained:

*There aren’t people you can share some of these things with and so I feel that I’m in a decent situation and just…I feel isolated at times.*

Similar to how one participant described “*filling a vessel that’s full of holes*,” another participant talked about the trajectory of grief throughout the caregiving process and how feelings changed for the caregiver over time:

*When this first happened, I really grieved a lot and I think I needed help around grieving the loss of a partner and a relationship…I’ve honestly lost a lot of my empathy. Like I just feel like the well has been dry…I think when this first started many years ago, I was better at putting myself in her shoes and trying to understand like how scared she was and the grief that she was feeling.*

Because the caregiver is often a similar age, considerations as to how their own healthcare needs could impact their ability to continue caregiving for the person with ADRD, adds an additional sense of fear, grief, and loss. Several caregivers expressed anxiety about who would be able to take on the caregiving role if they were no longer able to.

### Theme 5: entrapment of past and present stigma and discrimination

Caregivers had widely varying experiences of discrimination and acceptance around LGBT issues. Several reported a history of discrimination for both the person living with AD and themselves, that affected attitudes about coming out in the healthcare setting and contributed to the isolation experienced by many in the LGBT community. One participant shared:

*The things that she went through and the things that we had to do to go out and have some fun without having fear of losing jobs and stuff cause we were both teachers ……she was in the Marine Corps during the Korean conflict so she went through three investigations. Many of her friends got dishonorable discharges.*

However, this same caregiver reported strong support from both LGBT people and straight friends in a Mountain college-town:

*My gay community here in town, they’re part of that handful of people where all I have to do is make a phone call and they’ll be right here but I have the same amount of people on the straight end of that…of my extended family as well.*

Sometimes the caregiver was pleasantly surprised by the lack of discrimination:

*I’ll say in terms of just focusing a little bit on the gay and lesbian aspect, I expected to encounter more resistance or hesitation dealing with people because of, you know, we are in the South where she lives in the personal care home, you know, they are conservative and they are religious…but I found that people are so grateful that someone else has been…will step up and be the advocate for someone with dementia…and we’re just really glad that you’re taking care of this and we don’t have to…um…so I guess that’s made it easier.*

Negative experiences were also reported. One caregiver had moved from a very gay-friendly town to one that had better medical resources but a less inclusive community. This couple experienced intrusive questions from an in-home aide:

*There was one caregiver they gave us ... who actually (laughing) started reading Biblical passages and doing a couple of bizarre things and he (partner) was a little uncomfortable with that. I wasn’t comfortable with that although he was otherwise fine. We talked it over and decided that as long as he didn’t get too aggressive about this that we wouldn’t complain to management about him but eventually he sort of…he kept asking oh, well, do you have sex together…things like that*.

Within their relationships, some couples seemed to approach caregiving as many committed or married couples would with an assumption that the spouse would serve as the primary caregiver. In contrast, one caregiver pointed out that the lack of assumptions about marriage for LGBT couples provided an opportunity to define roles themselves. Another couple described the decision not to get married, since it would negatively impact Medicaid coverage and other services for the person living with AD.

## Discussion

This phenomenological study of the experiences of a geographically diverse group of caregivers for LGBT older adults with AD found several themes similar to the concerns of all caregivers: the importance of social support, financial security, and grief and bereavement support. However, LGBT status affected the experience of healthcare, raised concerns about acceptance in new care settings, and affected social support for some caregivers. While some caregivers reported being pleasantly surprised by the lack of discrimination that they experienced, they acknowledged that they feared discrimination when approaching a new care setting, and not all have openly disclosed their sexual orientation. These experiences have previously been shown to have a negative impact on health and health care [2, 3, 11]. For participants who had experienced having access to LGBT inclusive resources and LGBT affirming providers, they described the benefit of having access to these resources while navigating the other complexities of caregiving.

Facing discrimination in the healthcare setting adds to the burden of LGBT care. LGBT advocates have found that 56% of LGB and 70% of transgender older adults reported being denied or provided inferior health care because of their gender identity suggesting that the problem is widespread [9]. One important concern highlighted by a participant is that ADRD often requires that professional caregivers encounter people living with ADRD in private settings such as the home, where it may be impossible to hide intimate relationships and where people are at their most vulnerable, both emotionally and physically.

Even if the discrimination is never encountered, a life history of discrimination may affect care seeking by both the person living with ADRD and caregiver. Reluctance to disclose the relationship makes it more difficult to build relationships with clinicians and to seek out needed social support resources. Prior discrimination may also increase rates of depression and other health conditions for LGBT people, which will further complicate dementia care.

Although themes such as social support are present for all caregivers, we noted that many of the caregivers in this study reported small social networks, estrangement from the person living with ADRD or caregiver’s family, and few friends. This raises the question of whether LGBT caregivers tend to have smaller social networks compared to others. Since people living with ADRD and caregivers both report feeling isolated, this may be a case in which the intersectionality between having dementia and being LGBT compounds social isolation. A critical step to address this is to provide organizations who support caregivers of people with ADRD, with the training necessary to develop inclusive support groups that address issues that are specific to caregivers within the LGBT community.

Another critical step is to develop interventions that incorporate and address the challenges described by those closest to the issues, such that the resources offered to these individuals are tailored to real, rather than assumed immediate and long-term needs. Furthermore, these challenges should inform decisions leading to system change and strive to address current discrimination of LGBT people living with ADRD and their caregivers. Reviewing caregivers’ experiences of interacting with health care professionals and community organizations and resources through a phenomenological lens could offer LGBT people living with ADRD patients’ and caregivers’ experiences of interacting with health care professionals and community organizations and resources, a better understanding of these experiences; making improvements to address disparities experienced by these individuals has the potential to reduce heath disparities and expand access to high-quality care.

### Strengths and limitations

This paper has limitations. The sample size is small, predominately white, and did not to our knowledge include transgender people. Further research is needed to provide any comparisons between caregivers for LGBT people living with AD – including those living with related dementias since the caregiver experience may be different – and caregivers for heterosexual people living with AD, with a specific focus on including those who are diverse in race, ethnicity, sexual orientation, and gender identity. We have chosen to use the term LGBT in this paper because it most closely aligns with how the study participants self-identified in this study, as well as the cited works referenced in this paper [25].

## Conclusions

There is consensus in the literature that caregiving is emotionally, physically, and financially challenging. The concept of caregiving in health care, however, often takes a functional notion that suppresses the deep interpretive experiences that are the undercurrent of caregiving. Yet, research to investigate caregiving and literature surrounding the interaction between caregiving, AD, and the LGBT community remains significantly behind to meeting the current needs of this community. Taking a phenomenological approach has allowed us to see that caregiving is an interpretive event that has the potential to exert power over people and health outcomes [26]. Focusing on the unique experiences at the intersection between AD and LGBT communities helps us attend to various aspects of the caregiving experience and provides a method for discerning and describing this complex human experience through first person accounts. Our key findings were that in the LGBT context, caregivers experience many of the same challenges as all caregivers (stress, burnout, and financial issues) but for many caregivers each of these was specifically affected by the LGBT experience. Future caregiving interventions will need to address these intersectional issues, such as the impact of past and present discrimination on the health seeking behavior and health outcomes of LGBT older adults, as well as the difficulty of finding clinicians who are educated and welcoming of LGBT people living with AD and their caregivers. Failure to invest in resources and training within the healthcare system, that would protect and support LGBT people in the face of a serious illness, like AD, is a defining challenge that must be addressed.

## Data Availability

The datasets used and/or analyzed during the current study are available from the corresponding author on reasonable request.
